# Prevalence and related factors of sleep quality among Chinese undergraduates in Jiangsu Province: multiple models' analysis

**DOI:** 10.3389/fpsyg.2024.1343186

**Published:** 2024-04-10

**Authors:** Bin Hu, Wen Shen, Yun Wang, Qi Wu, Jiali Li, Xiaozhou Xu, Yaohui Han, Lishun Xiao, Dehui Yin

**Affiliations:** ^1^Key Laboratory of Human Genetics and Environmental Medicine, School of Public Health, Xuzhou Medical University, Xuzhou, China; ^2^Department of Respiratory Diseases, The Affiliated Huai'an Hospital of Xuzhou Medical University, The Second People's Hospital of Huai'an, Huai'an, Jiangsu, China; ^3^Department of Dermatology, The Affiliated Huai'an Hospital of Xuzhou Medical University, The Second People's Hospital of Huai'an, Huai'an, Jiangsu, China; ^4^Department of Biostatistics, School of Public Health, Xuzhou Medical University, Xuzhou, China; ^5^Center for Medical Statistics and Data Analysis, Xuzhou Medical University, Xuzhou, China

**Keywords:** sleep quality, undergraduates, risk factors, Connor-Davidson resilience scale, mobile phone addiction index, Pittsburgh Sleep Quality Index

## Abstract

**Background and aims:**

In China, a significant number of undergraduates are experiencing poor sleep quality. This study was designed to investigate the prevalence of poor sleep quality and identify associated factors among undergraduates in Jiangsu Province, China.

**Methods:**

A total of 8,457 participants were collected in 2022 using whole-group convenience sampling. The factors studied included basic demographics, family and social support, personal lifestyles, physical and mental health, mobile phone addiction index (MPAI), and the Connor-Davidson resilience scale (CD-RISC). The Pittsburgh Sleep Quality Index (PSQI) was used to assess sleep quality. Four models, including weighted multiple linear regression, binary logistic regression, weighted linear mixed model, and logistic regression with random effects, were applied to identify associated factors for sleep quality.

**Results:**

Of the 8,457 participants analyzed, 26.64% (2,253) were classified into the poor sleep quality group with a PSQI score >7. No significant relationship was found between sleep quality and gender, native place, economic level of family, physical exercise, dormitory light, dormitory hygiene, and amativeness matter. Risk factors for sleep quality identified by the four models included lower CD-RISC, higher MPAI, fourth grade or above, smoking, drinking, greater academic pressure, greater employment pressure, roommate sleeping late, noisy dormitory, poorer physical health status, poorer mental health status, and psychological counseling.

**Conclusions:**

These findings provide valuable insights for university administrators, enabling them to better understand the risk factors associated with poor sleep quality in undergraduates. By identifying these factors, administrators can provide targeted intervention measures and counseling programs to improve students' sleep quality.

## Introduction

Sleep is a fundamental physiological process that is essential for our overall health. It is also important for our cognitive, emotional, and physical systems (Baranwal et al., 2023). In recent years, sleep quality has declined among undergraduates and received attention from the public and academics (Wang et al., 2016; Ahmed et al., 2020; Li Y. et al., 2020). Unfortunately, a significant number of undergraduates are experiencing poor sleep quality. In some provinces of China, the proportion of undergraduates with poor sleep quality has crossed more than 30.0%; for example, the proportions in Jilin, Guizhou, and Hong Kong were 33.8%, 53.7%, and 57.5%, respectively (Suen et al., 2008; Li Y. et al., 2020; Zhou et al., 2022). Sleep quality among undergraduate students has also been poor in other countries such as Ethiopia (57.5%) and India (51.0%) (Lemma et al., 2012; Ghrouz et al., 2019). Some studies focusing on medical students also found a high prevalence of poor sleep quality−52.4% in Greece during COVID-19 (Eleftheriou et al., 2021), 76.0% in Saudi Arabia (Almojali et al., 2017), and 27.8% in Inner Mongolia Medical University of China (Wang et al., 2016). In Jiangsu Province, the status of sleep quality among undergraduates is still unknown, and it would be very interesting to investigate the prevalence of poor quality and identify the associated factors in this population.

Research has indicated that sleep deprivation can lead to metabolic disorders and negative effects, such as increased metabolite levels, which can lead to poor memory, poor concentration, lower academic performance, and emotional fluctuations (Durmer and Dinges, 2005; Curcio et al., 2006; Basner et al., 2013; Davies et al., 2014; Lo et al., 2016; St-Onge, 2017; Gerhardsson et al., 2019; Vaccaro et al., 2020). In addition, poor sleep quality can also increase the risk of developing mental health problems such as anxiety and depression (Almojali et al., 2017; Shao et al., 2020). Given the importance of sleep in maintaining wellbeing, it is essential to understand the factors that contribute to poor sleep quality among undergraduates. Additionally, it is also imperative to identify potential solutions to improve their sleep habits and reduce the negative effects.

Some studies have shown that sleep quality among Chinese undergraduates was a result of various factors. A cross-sectional study reported a few risk factors such as poor academic performance, interpersonal relationship, skipping breakfast, and higher grades (Wang et al., 2016). Another study found that being a freshman, alcohol use, gambling, exercising for more than 30 min a week on <1 day, satisfaction with parental love, and harmonious relationship with classmates were risk factors, while no learning pressure, never having self-injurious behaviors, and harmonious family relationship were protective factors (Li Y. et al., 2020). A gender-specific study in China identified some risk factors for poor sleep quality that were related to weak physical condition and smoking in males, while noisy dormitory, skipping breakfast, drinking coffee, playing games, bad physical condition, and severe academic stress led to poor sleep quality in females (Zhou et al., 2022). A cluster randomized-controlled trial in China showed that good dormitory sleep environments could maintain good sleep quality (Li et al., 2022). In addition, the use of electronic products was also related to poor sleep quality (Demirci et al., 2015; Li L. et al., 2020). For example, using mobile phones, computers, and other electronic products at night could affect the regulation of the sleep clock, prolong the time to fall asleep, and shorten sleep time. Besides, there is a significant amount of similar research evaluating related factors for sleep quality (Nyer et al., 2013; Bi et al., 2022; Peltz and Rogge, 2022; Xian et al., 2022). In summary, these factors that are associated with undergraduates' sleep quality can be classified into the following categories: demographics, personal lifestyle, family support, social support, dormitory environment, physical health, and mental health.

Because of the significant social and cultural diversity between different regions in China, previous findings may not fully represent the overall sleep characteristics of the Chinese population, especially undergraduates. Thus, our study not only aimed to examine the prevalence of poor sleep among undergraduates but also provide a comprehensive understanding of the factors that contribute to poor sleep quality among undergraduates in Jiangsu Province, China. The factors examined in this study also included mobile phone addiction and psychological resilience. By enhancing our understanding of these variables, this study may also help to develop effective strategies to improve sleep quality and ultimately promote undergraduates' life quality in China.

Special attention should be paid to the four statistical methods we utilized in association analysis. Except for the usual models of multiple linear regression and binary logistic regression, two mixed effect models were adopted such as linear mixed model and logistic random effect model. All these models are variations of the generalized linear mixed model (Bolker et al., 2009; Stroup, 2013). The mixed model is also known as the hierarchical model and contains both fixed and random effects. The random effects model represents the variability between different conditions or blocks, and they are incorporated into the model to account for the correlation between observations. In the present study, the four regression models were conducted with standard diagnostics, ensuring more accurate parameters and *p*-values. Various studies have utilized regression models to identify risk factors associated with poor sleep quality, but few strictly performed regression diagnostics, which can potentially result in biased results.

## Methods

### Participants and explanatory variables

A whole-group convenience sampling was performed in universities of Jiangsu Province using an electronic questionnaire on the Wenjuanxing platform (www.wjx.cn) from October to November 2022. We distributed the link and QR code of the questionnaire to the undergraduates via university teachers and a WeChat group. The questionnaire contained survey instructions explaining the online survey's purpose and significance. Each undergraduate took the survey voluntarily and had the option to withdraw at any moment. The confidentiality of data and student personal information was guaranteed. To ensure the quality and accuracy of data collection, the questionnaire was pre-tested to validate question effectiveness and comprehensibility. The sample size was thoroughly calculated based on the expected prevalence rate of sleep disorders among college students, according to the sample size calculation formula: $n=z_{\alpha }^{2}\times pq/d^{2}$. When the significance level is α = 0.05, *z*_α_ = 1.96. Let *p* be the expected prevalence rate and *q* = 1−*p*. Based on the preliminary survey results of this study, the estimated detection rate of sleep disorders among undergraduates is *p* = 16%. The allowable error is *d* = 0.1 × *p*. The estimated sample size needed for the survey is 2017 individuals.

A total of 8,587 undergraduates attended the questionnaire voluntarily, among which 130 participants with incomplete information were excluded, leading to a response rate of 98.49%. So, the final sample consisted of 8,457 participants (5,917 females and 2,540 males). Participants were all students living on campuses and enrolled in 14 majors, including literature, history, philosophy, law, economics, management, education, science, engineering, agronomy, military, art, medicine, and others.

The questions in the questionnaire were organized into continuous, categorical, and ordinal variables (see Table 1). In categorical and ordinal variables, some values were merged to avoid extremely small sample sizes in some subgroups. Participants who never smoked were classified as not smoking and those who smoked occasionally or often were classified as smoking. Participants who had never drunk alcohol were classified as not drinking, and those who drank alcohol occasionally or often were classified as drinking.

**Table 1:** The prevalence of poor sleep quality across basic demographic characteristics.

**Variables**	**Overall 8,457 (100)**	**Good 6,204 (73.36)**	**Bad 2,253 (26.64)**	**t/χ^2^**	***p*-value**
**Continuous variables**					
Age (year)	18.83 ± 1.05	18.79 ± 1.03	18.96 ± 1.09	6.74	**<0.001** ^**#**^
Height (cm)	167.52 ± 8.42	167.57 ± 8.39	167.39 ± 8.49	0.89	0.3722^#^
Weight (kg)	60.62 ± 12.67	60.78 ± 12.66	60.21 ± 12.69	1.83	0.0668^#^
BMI (kg/m^2^)	21.51 ± 3.75	21.56 ± 3.74	21.40 ± 3.76	1.72	0.0858^#^
PSQI	5.64 ± 3.60	3.90 ± 1.99	10.41 ± 2.60	107.90	**<0.001**
Subjective sleep quality	0.91 ± 0.64	0.74 ± 0.53	1.38 ± 0.68	40.50	**<0.001**
Sleep latency	1.23 ± 1.41	0.72 ± 0.95	2.63 ± 1.53	55.52	**<0.001**
Sleep duration	0.94 ± 0.88	0.71 ± 0.74	1.58 ± 0.90	40.85	**<0.001**
Habitual sleep efficiency	0.88 ± 1.36	0.44 ± 1.06	2.07 ± 1.39	50.60	**<0.001**
Sleep disturbances	0.96 ± 0.65	0.79 ± 0.55	1.44 ± 0.66	42.20	**<0.001**
Use of sleeping medication	0.11 ± 0.44	0.04 ± 0.25	0.29 ± 0.71	16.44	**<0.001**
Daytime dysfunction	0.60 ± 0.65	0.46 ± 0.56	1.01 ± 0.71	33.12	**<0.001**
CD-RISC	85.80 ± 18.26	87.29 ± 18.42	81.70 ± 17.15	12.99	**<0.001**
Self-improvement	28.64 ± 6.06	29.14 ± 6.12	27.28 ± 5.69	13.07	**<0.001**
Tenacity	43.83 ± 10.03	44.62 ± 10.12	41.65 ± 9.46	12.54	**<0.001**
Optimism	13.33 ± 3.12	13.53 ± 3.12	12.78 ± 3.05	9.98	**<0.001**
MPAI	44.74 ± 13.53	43.11 ± 13.20	49.22 ± 13.43	18.72	**<0.001** ^**#**^
Inability to control craving	19.10 ± 6.10	18.38 ± 5.86	21.09 ± 6.31	17.79	**<0.001**
Withdrawal and escape	7.80 ± 3.45	7.44 ± 3.37	8.78 ± 3.48	16.04	**<0.001** ^**#**^
Anxiety and feeling lost	8.93 ± 3.20	8.66 ± 3.19	9.66 ± 3.11	12.72	**<0.001** ^**#**^
Productivity loss	8.92 ± 3.05	8.63 ± 3.04	9.70 ± 2.95	14.33	**<0.001** ^**#**^
**Categorical variables**					
**Gender**					
Female	5,917 (70.00)	4,329 (69.78)	1,588 (70.48)	0.36	0.5488
Male	2,540 (30.00)	1,875 (30.22)	665 (29.52)		
**Medical major**					
No	7,233 (85.53)	5,291 (85.28)	1,942 (86.2)	1	0.3080
Yes	1,224 (14.47)	913 (14.72)	311 (13.8)		
**Native place**					
Rural	5,107 (60.39)	3,706 (59.74)	1,401 (62.18)	4	**0.0445**
Urban	3,350 (39.61)	2,498 (40.26)	852 (37.82)		
**Only child**					
No	5,187 (61.33)	3,743 (60.33)	1,444 (64.09)	9.7	**0.0018**
Yes	3,270 (38.67)	2,461 (39.67)	809 (35.91)		
**Smoking**					
No	8,027 (94.92)	5,956 (96.00)	2,071 (91.92)	56	**<0.001**
Yes	430 (5.08)	248 (4.00)	182 (8.08)		
**Drinking**					
No	5,775 (68.29)	4,364 (70.34)	1,411 (62.63)	45	**<0.001**
Yes	2,682 (31.71)	1,840 (29.66)	842 (37.37)		
**Roommate sleeping late**					
No	4,793 (56.67)	3,715 (59.88)	1,078 (47.85)	97	**<0.001**
Yes	3,664 (43.33)	2,489 (40.12)	1,175 (52.15)		
**Noisy dormitory**					
No	6,096 (72.08)	4,699 (75.74)	1,397 (62.01)	154	**<0.001**
Yes	2,361 (27.92)	1,505 (24.26)	856 (37.99)		
**Dormitory light**					
Not bright	6,028 (71.28)	4,496 (72.47)	1,532 (68.00)	16	**<0.001**
Bright	2,429 (28.72)	1,708 (27.53)	721 (32.00)		
**Dormitory hygiene**					
Good	2,652 (31.36)	1,829 (29.48)	823 (36.53)	38	**<0.001**
Poor	5,805 (68.64)	4,375 (70.52)	1,430 (63.47)		
**Amativeness matter**					
Never	4,059 (47.99)	3,034 (48.90)	1,025 (45.49)	8.2	**0.0163**
Ever	2,745 (32.46)	1,989 (32.06)	756 (33.56)		
Being in love	1,653 (19.55)	1,181 (19.04)	472 (20.95)		
**Psychological counseling**					
No	7,490 (88.57)	5,660 (91.23)	1,830 (81.23)	162	**<0.001**
Yes	967 (11.43)	544 (8.77)	423 (18.77)		
**Ordinal variables**					
**Grade** ^*^					
First year	5,140 (60.78)	3,929 (63.33)	1,211 (53.75)	65	**<0.001**
Second year	2,496 (29.51)	1,714 (27.63)	782 (34.71)		
Third year	357 (4.22)	251 (4.05)	106 (4.70)		
Fourth year or more	464 (5.49)	310 (5.00)	154 (6.84)		
**Father's education level**					
Middle school or less	5,834 (68.89)	4,217 (67.97)	1,617 (71.77)	11	**0.0036**
Junior college education	1,369 (16.19)	1,041 (16.78)	328 (14.56)		
Undergraduate or more	1,254 (14.83)	946 (15.25)	308 (13.67)		
**Mother's education level**					
Middle school or less	6,256 (73.97)	4,568 (73.63)	1,688 (74.92)	1.7	0.4236
Junior college education	1,229 (14.53)	919 (14.81)	310 (13.76)		
Undergraduate or more	972 (11.49)	717 (11.56)	255 (11.32)		
**Family economic level**					
Good	725 (8.57)	568 (9.16)	157 (6.97)	53	**<0.001**
Medium	6,619 (78.27)	4,914 (79.21)	1,705 (75.68)		
Poor	1,113 (13.16)	722 (11.64)	391 (17.35)		
**Monthly living expense (CNY)**					
<1,000	1,030 (12.18)	689 (11.11)	341 (15.14)	26	**<0.001**
1,000–2,000	6,205 (73.37)	4,623 (74.52)	1,582 (70.22)		
≥2,000	1,222 (14.45)	892 (14.38)	330 (14.65)		
**Physical exercise**					
≤ 1 per month	1,391 (16.45)	925 (14.91)	466 (20.68)	42	**<0.001**
1–3 times per week	5,681 (67.18)	4,224 (68.09)	1,457 (64.67)		
4–7 times per week	1,385 (16.38)	1,055 (17.01)	330 (14.65)		
**Academic pressure**					
No	378 (4.47)	295 (4.75)	83 (3.68)	158	**<0.001**
Normal	6,052 (71.56)	4,640 (74.79)	1,412 (62.67)		
Great	2,027 (23.97)	1,269 (20.45)	758 (33.64)		
**Employment pressure**					
No	1,421 (16.80)	1,135 (18.29)	286 (12.69)	173	**<0.001**
Normal	4,281 (50.62)	3,295 (53.11)	986 (43.76)		
Great	2,755 (32.58)	1,774 (28.59)	981 (43.54)		
**Relationship with classmates**					
Harmonious	5,877 (69.49)	4,523 (72.9)	1,354 (60.1)	140	**<0.001**
Ordinary	2,453 (29.01)	1,615 (26.03)	838 (37.19)		
Poor	127 (1.50)	66 (1.06)	61 (2.71)		
**Physical health status**					
Good	3,668 (43.37)	2,987 (48.15)	681 (30.23)	308	**<0.001**
Ordinary	4,395 (51.97)	3,033 (48.89)	1,362 (60.45)		
Poor	394 (4.66)	184 (2.97)	210 (9.32)		
**Mental health status**					
Good	4,209 (49.77)	3,503 (56.46)	706 (31.34)	521	**<0.001**
Ordinary	3,787 (44.78)	2,499 (40.28)	1,288 (57.17)		
Poor	461 (5.45)	202 (3.26)	259 (11.5)		

^*^Grade is the term used for undergraduate student years in China.

^#^Denotes that the groups satisfy homoscedasticity and others do not.

*p*-values <0.05 are given in bold.

### Pittsburgh Sleep Quality Index

The Pittsburgh Sleep Quality Index (PSQI) (Buysse et al., 1989; Chen et al., 1999) was adopted to assess the sleep quality of undergraduates by translating it into a Chinese version. PSQI includes 19 self-rated questions, and only the first 18 entries are used to calculate scores. These questions can be grouped into seven dimensions that can evaluate sleep quality in the last month, including subjective sleep quality, sleep latency, sleep duration, habitual sleep efficiency, sleep disturbances, use of sleeping medication, and daytime dysfunction. The final PSQI score was calculated by summing the scores from these seven dimensions. The total score ranges from 0 to 21 (0–3 for each component), with higher values indicating poor sleep quality. A final PSQI score of more than 7 indicates poor sleep quality and a score of <7 represents good sleep quality. For the PSQI conducted in this study, Cronbach's alpha was 0.877.

### Mobile phone addiction index

The mobile phone addiction index (MPAI) (Bianchi and Phillips, 2005), which was already translated into Chinese and validated for the Chinese population (Li L. et al., 2020), was used to evaluate mobile phone addiction. The MPAI consists of 17 items quantifying four dimensions, including the inability to control craving, withdrawal and escape, anxiety and feeling lost, and productivity loss. Scores range from 1 (not at all) to 5 (always) for each item. The higher total scores indicate a higher level of mobile phone addiction. The Cronbach's alpha for the MPAI of the present study was 0.925.

### Connor-Davidson resilience scale

The present study employed the Connor-Davidson resilience scale (CD-RISC) (Connor and Davidson, 2003) to measure the psychological resilience of undergraduates, which reflects how the participants felt over the last month. This scale has been employed in various populations and has also been translated into Chinese (Xu et al., 2022). The CD-RISC consists of 25 items, and each item's score ranges from 0 to 4, representing not true at all, rarely true, sometimes true, usually true, and true almost all the time, respectively. The final CD-RISC score was obtained by summing each score of the items, which ranged from 0 to 100, with higher scores indicating greater resilience. The Cronbach's alpha for the CD-RISC of the present study was 0.969.

### Statistical analysis

The software R version 4.3.0 was used for statistical analysis. Extreme values of continuous variables were identified by the interquartile range (IQR) method. The upper and lower fences were defined as *Q*3+2*IQR* and *Q*1 − 2*IQR*. Values below (resp., above) the lower (resp., upper) fence were replaced with the lower (resp., upper) fence. For continuous variables, their central and variation trends were described by mean and standard deviation (SD), i.e., $\bar{x}\pm s$, and the means between two (resp., or more) groups were compared by *t*-test (resp. ANOVA) when homoscedasticity was satisfied, otherwise, Welch's *t*-test (resp., Welch's ANOVA) was employed. A *post-hoc* test was performed using Tukey's method when homoscedasticity was satisfied; otherwise, the Games-Howell test was performed. In this study, homoscedasticity was measured using Levene's test. The categorical and ordinal variables were described by frequency and constituent ratio, and the differences between groups were compared by Chi-square test. The significant level was set as 0.05.

### Statistical models

#### Multiple linear regression model

The associated factors for undergraduate sleep quality were identified using a linear regression model with the continuous variable PSQI score as the dependent variable, other variables as explanatory variables, and ordinal variables as continuous. The standard regression diagnostics (Kabacoff, 2015) were processed after the multiple linear regression model (MLR) was implemented with the following steps: (a) Samples containing extreme values, including outliers, high leverage points, and influential points were removed. The outliers were detected if the Bonferroni adjusted *p*-value of the corresponding absolute studentized residuals was significant; the high leverage points were identified via the hat statistics; the influential points were determined by Cook's distance. In our model, 497 samples were removed in this step. (b) The independence of residuals was checked using the Durbin–Watson's test. In our model, independence was satisfied. (c) Multicollinearity between explanatory variables was detected through variance inflation factor (VIF). In our model, the VIF values of BMI, weight (kg), and height (cm) were all more than 4, which indicated that multicollinearity exists. Hence, weight and height were removed since BMI contains information about both. (d) The normality and homoscedasticity of residuals were checked. Our detection indicated that homoscedasticity was not satisfied. Therefore, weighted MLR was utilized to solve the heteroscedasticity problem, with weights inversely proportional to the variance of the dependent variable. The absolute values of the residuals were regressed against the fitted values, and the resulting fitted values were squared to provide the desired estimate of the variance. (e) The linearity between dependent variables and explanatory variables was checked. The performance of linearity, homoscedasticity, normality, and extreme values after diagnostics is shown in Supplementary Figure S1. Visual inspection did not reveal any obvious deviations from linearity or normality. It is noteworthy that non-normality is less likely to be a problem when other assumptions are met since Aitken's theorem shows that the regression coefficients obtained from weighted MLR are also the best linear unbiased estimator without the assumption of normality (Hansen, 2022). The linear trend test for a continuous variable was performed by transforming it into a four-valued ordinal variable by quartiles in the weighted MLR model.

#### Binary logistic regression model

Odds ratios (ORs) of factors associated with poor sleep quality were performed using a binary logistic regression model (BLR). The dependent variable was set as good (coded as 0) and poor (coded as 1) sleep quality based on whether the PSQI score was <7, and other variables were treated in the same way as MLR. The diagnostics (Kabacoff, 2015) were processed with the following steps: (a) Extreme values were detected by the same method as MLR and 408 samples were removed. (b) Multicollinearity was detected by VIF, and weight and height were also excluded. (c) The independence of observations was performed using Durbin–Watson's test, and this assumption was satisfied. (d) The linearity between the log odds and explanatory variables was checked by regressing the log odds against the explanatory variables. The performance of linearity and extreme values after diagnostics is shown in Supplementary Figure S2. (e) Overdispersion and events per variable (EPV) were checked (Peduzzi et al., 1996). Overdispersion was accessed by the Chi-square test, but it did not exist in our model. There were 28 explanatory variables, including dummy variables, 1,897 samples with good sleep quality, and 6,152 samples with poor sleep quality. Thus, the EPV principle was satisfied. The ratio of these two outcome events was ~1:3; therefore, it can be considered as approximately balanced data. The linear trend test was performed in the same way as the aforementioned method.

#### Linear mixed model

The linear mixed model (LMM) is an extension of the MLR to allow both fixed and random effects, and it is particularly used in hierarchical analysis (Stroup, 2013). The package “lmerTest” was adopted to implement LMM. In LMM, the continuous variable PSQI served as the dependent variable. The candidate random effect terms were checked by the likelihood ratio test. As random effects, we had intercepts for grade, smoking, drinking, physical health status, mental health status, academic pressure, employment pressure, and psychological counseling, as well as random slopes for the effect of relationship with classmates among academic pressure and employment pressure. Other variables, excluding weight and height due to multicollinearity, were set as fixed effects. No outliers were detected, and the homoscedasticity was violated. Therefore, the weighted LMM was adopted, and the weights were determined in the same way as the weighted MLR. The independence was checked using Durbin–Watson's test, and the results were satisfied. The performance of linearity, homoscedasticity, normality, and extreme values after diagnostics is shown in Supplementary Figure S3. Visual inspection did not reveal any obvious deviations from linearity or normality.

#### Logistic regression with random effects

The logistic regression with random effects model (LRRE) is a type of generalized linear mixed model. The dependent variable was set as BLR. We had random intercepts for grade, smoking, drinking, physical health status, mental health status, academic pressure, employment pressure, and psychological counseling, as well as random slopes for the effect of the relationship with classmates against employment pressure and academic pressure. Other variables, excluding weight and height due to multicollinearity, were set as fixed effects. A likelihood ratio test was performed to check the significance of random effects. No outliers were detected. Independency, linearity, and overdispersion were checked in the same way as BLR, and all assumptions were satisfied. The performance of linearity after diagnostics is shown in Supplementary Figure S4.

## Results

### Prevalence of poor sleep quality

The Cronbach's alpha for the questionnaire of this study was 0.911, which indicates the internal consistency of the questionnaire was good. The sample consisted of 69.97% male and 30.03% female. Out of the 8,457 undergraduates, 6,204 (73.36%) reported having good sleep quality while 2,253 (26.64%) experienced poor sleep quality, based on the PQSI threshold value of 7. The mean PSQI score of all participants was 5.64 ± 3.60. The prevalence of poor sleep quality in undergraduates is shown in Table 1, and the PSQI score across different characteristics is shown in Supplementary Table S1. There was no significant difference between male (5,917) and female (2,540) students in height, weight, BMI, medical major, and non-medical major in terms of PSQI score or sleep quality. Older students and those in higher grades had a higher prevalence of poor sleep quality compared with their counterparts who were younger and in lower grades. The prevalence of poor sleep quality among students from rural areas, only-child families, and fathers with low education levels was nearly 30% higher than among students from urban areas, having siblings and fathers with high education levels. Although the PSQI scores of the three levels of mothers' education were significantly different via Welch's ANOVA, the *post hoc* result did not show the difference, and the constituent ratios between students with good and poor sleep quality were not significantly different. Approximately 80% of the students with either good or poor sleep quality belonged to families with medium economic levels. Similarly, more than 70% of the students with good or poor sleep quality reported a monthly living expense between 1,000 to 2,000 CNY. In terms of dormitory and personal lifestyle, the constituent ratios between students with good and poor sleep quality were all significantly different, and the corresponding PSQI scores were also significantly different. The worse these parameters were, the higher the prevalence of poor sleep quality.

Undergraduates with poor sleep quality had higher PSQI scores across all seven components compared to those with good sleep quality. The mean PSQI score for undergraduates with poor sleep quality was 10.41, which was significantly higher than their counterparts with good quality sleep (*p* < 0.001). The mean scores of CD-RISC and its three components for students with poor sleep quality were all significantly lower than those with good sleep quality (*p* < 0.001). The MPAI situation was the opposite.

### Results of weighted MLR and BLR

The Akaike Information Criterion (AIC) of MLR and weighted MLR were 38,170.2 and 38,103.3, respectively, which indicates that weighted MLR improved the bias caused by heteroscedasticity in MLR. Table 2 presents the factors associated with PSQI scores, which were identified by weighted MLR, and the risk factors for poor sleep quality identified through BLR. The variables age, MPAI, grade, smoking, drinking, academic pressure, employment pressure, relationship with classmates, roommate sleeping late, noisy dormitory, physical health status, mental health status, and psychological counseling exhibited significant positive associations with the PSQI scores. On the other hand, BMI, CD-RISC, medical major, and father's education level demonstrated significant negative correlations with the PSQI scores. Barring BMI and relationship with classmates, the association between all other factors and PSQI was linear.

**Table 2:** Risk factors for sleep quality identified by weighted MLR and BLR.

**Variable**	**Weighted MLR**			**BLR**		
	***b*** **, 95%CI**	***p*** **-value**	***p*** **for trend**	**OR, 95%CI**	***p*** **-value**	***p*** **for trend**
Age	0.139 (0.056, 0.222)	**0.001**	**0.011**	1.080 (0.996, 1.170)	0.062	**–**
BMI	−0.021 (−0.037, −0.005)	**0.009**	0.090	0.959 (0.943, 0.975)	**<0.001**	**<0.001**
CD-RISC	−0.017 (−0.020, −0.013)	**<0.001**	**<0.001**	0.986 (0.982, 0.990)	**<0.001**	**<0.001**
MPAI	0.044 (0.040, 0.049)	**<0.001**	**<0.001**	1.036 (1.031, 1.041)	**<0.001**	**<0.05** ^**L, Q**^
Grade	0.177 (0.066, 0.287)	**0.002**	**–**	1.178 (1.059, 1.310)	**0.003**	0.104
MM: yes	−0.335 (−0.509, −0.162)	**<0.001**	**–**	0.675 (0.559, 0.813)	**<0.001**	**–**
OC: yes	−0.096 (−0.228, 0.035)	0.150	**–**	0.811 (0.710, 0.926)	**0.002**	**–**
FEL	−0.162 (−0.272, −0.053)	**0.004**	**0.003**	0.777 (0.690, 0.873)	**<0.001**	**<0.001**
MEL	0.078 (−0.041, 0.197)	0.198	**–**	1.214 (1.072, 1.375)	**0.002**	**0.009**
Smoking: yes	0.953 (0.629, 1.277)	**<0.001**	**–**	1.568 (1.195, 2.052)	**0.001**	**–**
Drinking: yes	0.382 (0.245, 0.519)	**<0.001**	**–**	1.255 (1.101, 1.431)	**0.001**	**–**
AP	0.319 (0.184, 0.455)	**<0.001**	**<0.001**	1.177 (1.036, 1.337)	**0.012**	**0.034**
EP	0.183 (0.087, 0.280)	**<0.001**	**<0.001**	1.226 (1.114, 1.349)	**<0.001**	**<0.001**
RWC	0.196 (0.061, 0.332)	**0.004**	0.066	1.021 (0.906, 1.151)	0.73	**–**
RSL: yes	0.255 (0.120, 0.390)	**<0.001**	**–**	1.141 (1.000, 1.301)	**0.049**	**–**
ND: yes	0.554 (0.393, 0.716)	**<0.001**	**–**	1.543 (1.332, 1.787)	**<0.001**	**–**
PHS	0.459 (0.331, 0.587)	**<0.001**	**<0.001**	1.331 (1.178, 1.505)	**<0.001**	**<0.001**
MHS	0.769 (0.637, 0.902)	**<0.001**	**<0.001**	2.028 (1.796, 2.292)	**<0.001**	**<0.001**
PC: yes	0.651 (0.442, 0.860)	**<0.001**	**–**	1.383 (1.169, 1.633)	**<0.001**	**–**

MM, medical major, OC, only child, FEL, father's education level, MEL, mother's education level, AP, academic pressure, EP, employment pressure, RWC, relationship with classmates, RSL, roommate sleeping late, ND, noisy dormitory, PHS, physical health status, MHS, mental health status, and PC, psychological counseling.

Superscripts L and Q represent linear trend and quadratic trend, respectively. *p*-values <0.05 are given in bold.

**Figure 1:**
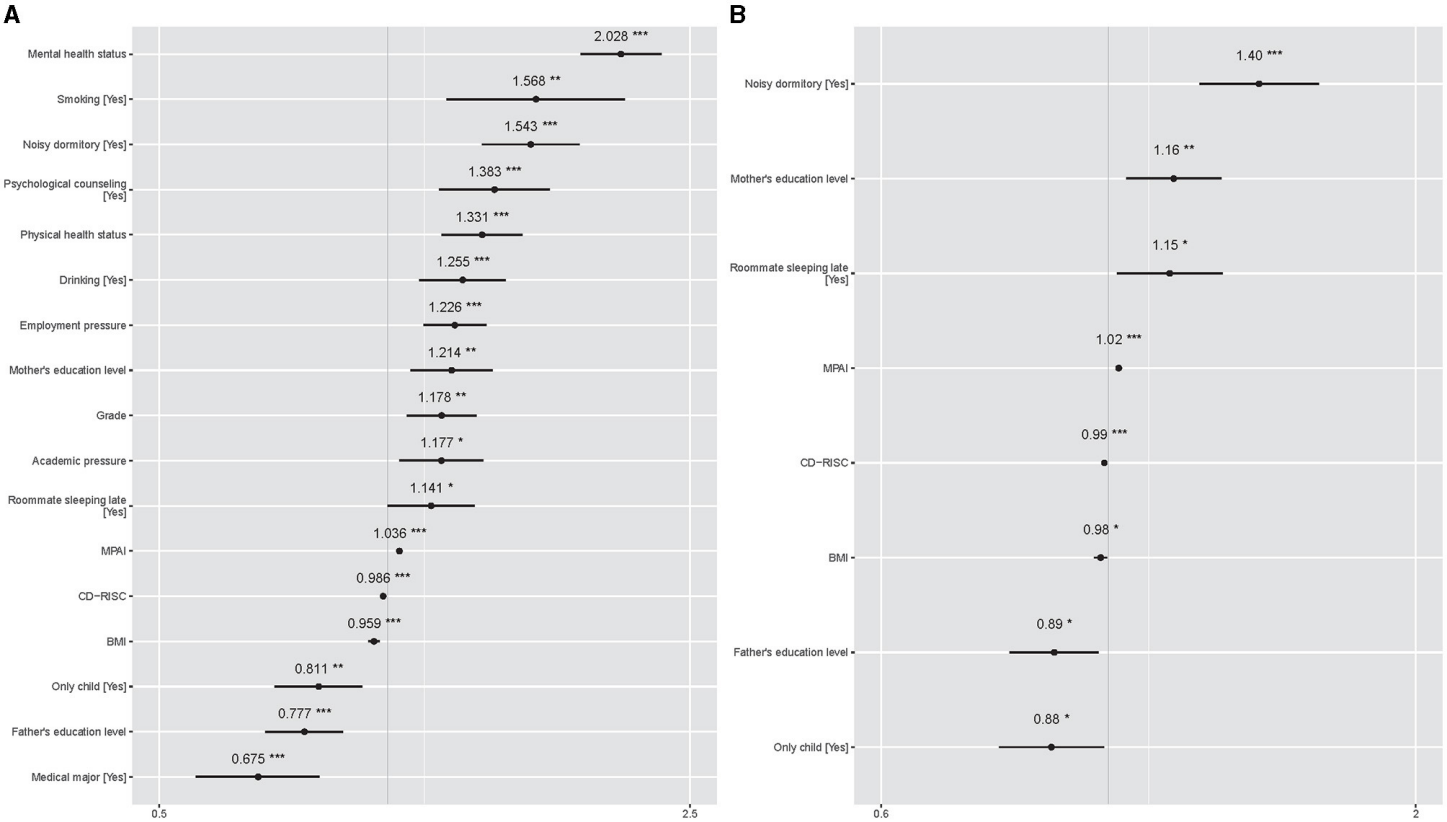
Sorted ORs of significant factors for undergraduate poor sleep quality. The gray vertical solid line represents OR =1, and the short black horizontal solid line represents 95% CIs of ORs. Symbol * denotes *p* < 0.05, ** denotes *p* < 0.01, and *** denotes *p* < 0.001. **(A)** Comes from the results of BLR. **(B)** Comes results of LRRE.

The results of BLR showed that students with higher BMI (OR = 0.959, *p* < 0.001), CD-RISC (OR = 0.986, *p* < 0.001), and father's education level (OR = 0.777, *p* < 0.001), had a decreased risk of poor sleep quality. Students with higher MPAI (OR = 1.036, *p* < 0.001), higher grades (OR = 1.178, *p* = 0.003), higher mother's education level (OR = 1.214, *p* = 0.002), greater academic pressure (OR = 1.177, *p* = 0.012), greater employment pressure (OR = 1.226, *p* < 0.001), worse physical health status (OR = 1.331, *p* < 0.001), and worse mental health status (OR = 2.028, *p* < 0.001) had an increased risk of poor sleep quality. Students who majored in medicine (OR = 0.675, *p* < 0.001) and were the only child in the family (OR = 0.811, *p* = 0.002) had a lower risk of poor sleep quality than those who did not major in medicine and were not the only child in the family. Smoking (OR = 1.568, *p* < 0.001), drinking (OR = 1.255, *p* < 0.001), and receiving psychological counseling (OR = 1.383, *p* < 0.001) were significantly associated with poor sleep quality. Roommates sleeping late (OR = 1.141, *p* = 0.049) and noisy dormitory (OR = 1.543, *p* < 0.001) were shown to be risk factors for poor sleep quality. The OR of mental health status was the highest, followed by smoking, noisy dormitory, psychological counseling, physical health status, and drinking. The sorted ORs of significant factors for undergraduate poor sleep quality can be found in Figure 1. Linear trend test showed that MPAI had both significant linear and quadratic trends with respect to poor sleep quality, but no substantial distinction was observed between the two trends on visual inspection (see Supplementary Figure S4). Besides, there was also no linear trend between grade and poor sleep quality (*p* = 0.104). The receiver operating characteristic (ROC) curve of BLR can be found in Supplementary Figure S5, and the area under the curve (AUC) was 0.776.

### Results of weighted LMM and LRRE

**Figure 2:**
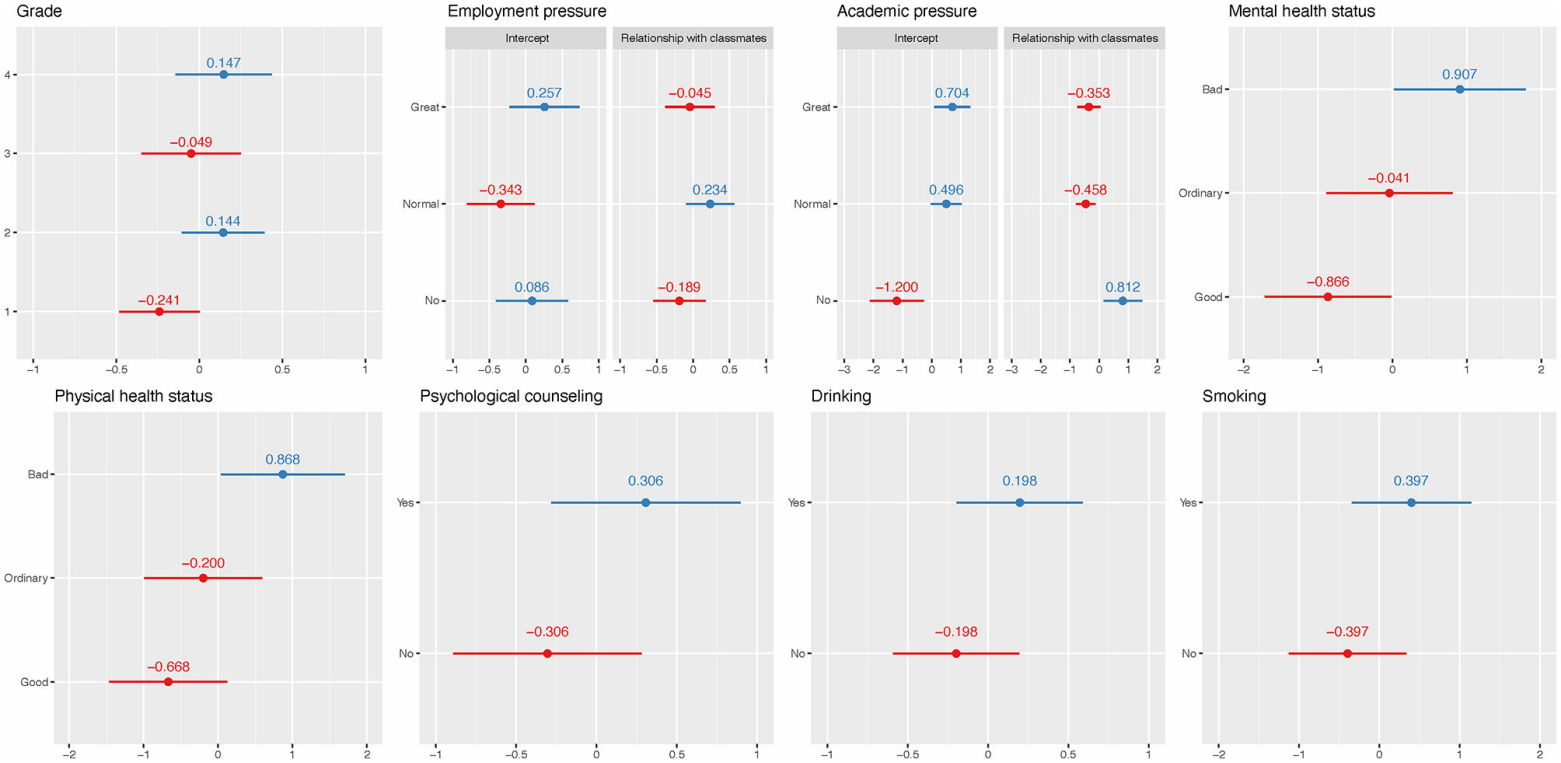
Random effects of weighted LMM. The red and blue solid dots represent negative and positive effects on PSQI scores, respectively. The short horizontal solid lines represent 95% CI.

The AICs of LMM and weighted LMM were 43,857.1 and 43,554.2, respectively, which indicates that weighted LMM improved the bias caused by heteroscedasticity in LMM. Table 3 shows fixed and random effects of associated PSQI score factors identified by weighted LMM and related factors for poor sleep quality identified by LRRE. The intraclass correlation coefficients (ICCs) of the two models were 0.123 and 0.130, suggesting that 12.3% and 13.0% of the total variation in the responses were explained by subgroups. The fixed effects of age (*b* = 0.142, *p* = 0.002) and MPAI (*b* = 0.043, *p* < 0.001) exhibited a significant positive association with respect to PSQI scores, while the fixed effect of CD-RISC was the opposite (*b* = −0.016, *p* < 0.001). Students with a roommate sleeping late (*b* = 0.303, *p* < 0.001) and in a noisy dormitory (*b* = 0.582, *p* < 0.001) had significantly higher PSQI scores at an average level compared to those without a roommate sleeping late and in a noisy dormitory. Except for age, all significant continuous factors displayed a linear trend with the PSQI score. However, an analysis of the trend graph between age and PSQI score revealed that the quadratic trend was approximately close to linearity (see Supplementary Figure S6). The random effects of weighted LMM explained 15.62% of the total variation. The detailed random effect values of weighted LMM can be found in Figure 2. For random intercepts, being in the second and fourth grades, having poor mental and physical health status, receiving psychological counseling, smoking, and drinking had the effect of increasing PSQI scores. The random slopes of relationships with classmates varied across the three levels of employment pressure and academic pressure. However, the random effects of the relationship with classmates and employment pressure were not significant. In the subgroup of students without academic pressure, the intercept was −1.200, and the slope of the relationship with classmates was 0.812, suggesting that a poor relationship with classmates had a positive effect on increasing PSQI scores. In the subgroups of students with normal and great academic pressure, the intercepts were 0.496 and 0.704, respectively, indicating that these two subgroups had higher average PSQI scores compared to the subgroups of students without academic pressure. However, the slopes of relationship with classmates across the two subgroups were −0.458 and −0.353, demonstrating that a harmonious relationship with classmates had a positive effect on increasing the PSQI scores.

**Table 3:** Fixed and random effects of factors for sleep quality by weighted LMM and LRRE.

**Variable**	**Weighted LMM**			**LRRE**		
**Fixed effect**	***b*** **, 95% CI**	***p*** **-value**	***p*** **for trend**	**OR, 95% CI**	***p*** **-value**	***p*** **for trend**
Intercept	3.605 (0.690, 6.519)	**0.024**	**–**	0.155 (0.025, 0.955)	**0.044**	**–**
Age	0.142 (0.054, 0.231)	**0.002**	**0.014** ^**Q**^	1.071 (0.999, 1.148)	0.053	**–**
BMI	−0.011 (−0.029, 0.007)	0.231	**–**	0.983 (0.969, 0.998)	**0.022**	**0.010**
CD-RISC	−0.016 (−0.019, −0.012)	**<0.001**	**<0.001**	0.992 (0.988, 0.995)	**<0.001**	**<0.001**
MPAI	0.043 (0.038, 0.049)	**<0.001**	**<0.001**	1.024 (1.020, 1.028)	**<0.001**	**<0.001**
OC: yes	−0.083 (−0.233, 0.067)	0.277	**–**	0.880 (0.782, 0.990)	**0.034**	**–**
FEL	−0.101 (−0.224, 0.023)	0.111	**–**	0.886 (0.801, 0.979)	**0.017**	**0.033**
MEL	0.065 (−0.069, 0.2)	0.340	**–**	1.159 (1.041, 1.290)	**0.007**	**0.007**
RSL: yes	0.303 (0.148, 0.459)	**<0.001**	**–**	1.149 (1.020, 1.294)	**0.022**	**–**
ND: yes	0.582 (0.394, 0.770)	**<0.001**	**–**	1.405 (1.229, 1.606)	**<0.001**	**–**
**Random effect**	**Variance (4.004)**	**–**	**–**	**Variance (0.856)**	**–**	**–**
Grade: intercept	0.047	**<0.001**	**–**	0.015	**0.003**	**–**
EP: intercept	0.127	**<0.001**	**–**	0.086	**0.007**	**–**
RWC	0.063	0.210	**–**	0.040	0.128	**–**
Drinking: intercept	0.081	**<0.001**	**–**	0.016	**0.009**	**–**
Smoking: intercept	0.332	**<0.001**	**–**	0.084	**<0.001**	**–**
AP: intercept	1.176	**0.007**	**–**	0.182	**0.047**	**–**
RWC	0.545	**0.030**	**–**	0.130	0.250	**–**
PHS: intercept	0.636	**<0.001**	**–**	0.080	**<0.001**	**–**
MHS: intercept	0.802	**<0.001**	**–**	0.182	**<0.001**	**–**
PC: intercept	0.195	**<0.001**	**–**	0.041	**<0.001**	**–**
**Residual**	16.687		**–**	3.29	**–**	**–**
**ICC**	0.123		**–**	0.130	**–**	**–**

MM, medical major; OC, only child; FEL, father's education level; MEL, mother's education level; AP, academic pressure; EP, employment pressure; RWC, relationship with classmates; RSL, roommate sleeping late; ND, noisy dormitory; PHS, physical health status; MHS, mental health status; PC, psychological counseling; and ICC, intraclass correlation coefficient.

Superscript Q represents the quadratic trend. *p*-values <0.05 are given in bold.

**Figure 3:**
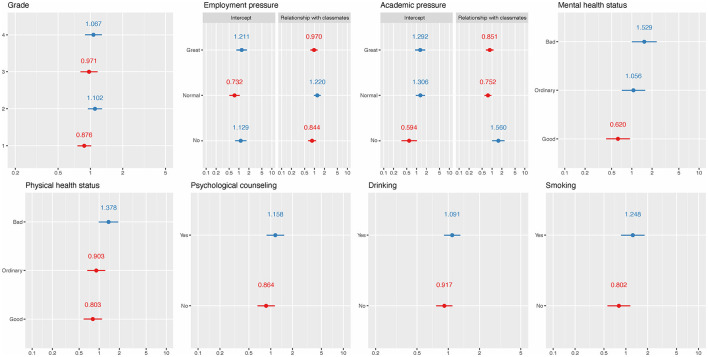
ORs for random effects of LREE. The red and blue solid dots represent ORs that are <1 and >1, respectively. The short horizontal solid lines represent 95% CI.

For the fixed effect results of LRRE, higher BMI (OR = 0.983, *p* = 0.022) and CD-RISC (OR = 0.992, *p* < 0.001) had a decreased risk of poor sleep quality. On the contrary, students with higher MPAI (OR = 1.024, *p* < 0.001) and mother's education level (OR = 1.159, *p* = 0.007) had an increased risk of poor sleep quality. Being the only child was a protective factor of poor sleep quality compared to not being the only child (OR = 0.88, *p* = 0.034). All significant continuous factors had a linear trend with respect to poor sleep quality. A higher risk was also found for students with roommates sleeping late (OR = 1.149, *p* = 0.022) and noisy dormitories (OR = 1.405, *p* < 0.001). These results are consistent with previous BLR results. In fixed effects, noisy dormitory had the highest OR, followed by mother's education level and roommates sleeping late. The sorted ORs can be found in Figure 1. The random effects explained 20.65% of the total variation when the dataset was fitted by LRRE and their ORs, which can be found in Figure 3. It was found that the variability in students' relationships with classmates (random slopes) did not significantly differ across the three levels of employment pressure and academic pressure. This suggests that students' relationships with classmates were consistent regardless of the degree of pressure experienced. The effects of most random intercepts were consistent with the corresponding counterpart of weighted LMM. Being in the second and fourth grades, experiencing “great” and “not having” employment pressure, poor and normal levels of mental health status, poor physical health status, receiving psychological counseling, smoking, and drinking had a positive effect on poor sleep quality. The ROC curve of LRRE can be found in Supplementary Figure S7, and the AUC was 0.720.

## Discussion

This study investigated the prevalence and associated factors of sleep quality among undergraduates in Jiangsu Province, China. The results showed a mean PSQI score of 5.64 ± 3.60 and a prevalence of poor sleep quality of 26.64%, which is consistent with another study on medical college students in China, which showed the prevalence was 27.80% (Wang et al., 2016) but lower than the results obtained in Ethiopia (55.8%) and Taiwan (33.80%) (Kang and Chen, 2009; Lemma et al., 2012).

No significant association was found between gender and sleep quality through demographics and the four regression models. Similar results were reported in previous studies (Lund et al., 2010; Wang et al., 2016; Li Y. et al., 2020). The study also found no significant correlation between sleep quality and native place, family economic level, physical exercise, dormitory light, dormitory hygiene, and amativeness matter through the four regression models. Previous studies (Lund et al., 2010; Wang et al., 2016; Li Y. et al., 2020) also reported that there was no association between sleep quality and family economic level, physical exercise, and amativeness matter. Some studies showed that sleep quality might be influenced by native places (Tang et al., 2017), but their cutoff values of PSQI score was not seven, and the population was not undergraduates. Our study found that there is no significant correlation between sleep quality and dormitory light brightness or dormitory hygiene; these factors were included in the cluster randomized-controlled trial described in research (Li et al., 2022) to explore comprehensive environmental influences on sleep quality. The rationale for this inclusion is based on the hypothesis that environmental factors, beyond personal habits or demographic characteristics, have a significant impact on sleep quality. Although our findings and the intervention in the cited study did not demonstrate a direct impact of these factors on sleep quality, including them in the analysis will help to provide a holistic view of the environmental influences on sleep.

Since the dependent variable PSQI score in weighted MLR and weighted LMM was continuous, while the dependent variable in BLR and LRRE was dichotomous, the associated factors identified by the two types of models may differ. For example, age was significantly positively associated with PSQI score in weighted MLR and weighted LMM but was not significant in BLR and LRRE. Being the only child and mother's education level were significant in BLR and LRRE but were not in weighted MLR and weighted LMM. This may be because age can affect the PSQI score, but it has a limited impact on PSQI in the bounded range (Basner et al., 2013; Demirci et al., 2015).

In the present study, the BLR and LRRE models identified significant associations between only child status and mother's education level with sleep quality. The effects of these factors appeared to be amplified in the dichotomous models (BLR and LRRE) than in the other models, suggesting that these variables may have a more sensitive response to categorical sleep quality measures. This phenomenon may reflect the complex relationship between these sociodemographic characteristics and individuals' daily behavioral patterns. Specifically, students who are the only child may receive more attention and resources within the family and also be subject to higher expectations and pressures from their parents, both of which may affect sleep quality.

Higher levels of maternal education may also imply better cognitive stimulation and family economic conditions, which indirectly affect the child's sleep status. On the other hand, these significant differences may be related to the family's socio-economic status and cultural background, which also affect undergraduates' performance in school or social activities and stress levels, and ultimately affect sleep quality. There was a difference in sleep quality between the only child and non-only child subgroups (see Table 1), but the difference in PSQI score was small (see Supplementary Table S1), resulting in the linear model not identifying it after adjusting for other factors. The same situation also applies to the mother's education level, in which the three levels had no difference in the PSQI score. BMI and the father's education levels were not significant in the weighted LMM but were significant in the other three models.

Relationship with classmates was significant in weighted MLR and had significant random slopes in weighted LMM but was not significant in BLR and LRRE. Some inconsistencies between weighted MLR and weighted LMM, BLR, and LRRE may be due to the different samples included. Three kinds of extreme values were removed from the weighted MLR and BLR, while LMM and LRRE had relatively few methods to detect extreme values. Weighted LMM and LRRE contained all samples since no extreme values were found in the diagnostic step.

The four regression models identified several common risk factors for sleep quality, including lower CD-RISC, higher MPAI, being in fourth grade or above, smoking, drinking, greater academic pressure, having no or great employment pressure, roommate sleeping late, noisy dormitory, poorer physical health status, poorer mental health status, and psychological counseling. Most of these results are consistent with previous studies. CD-RISC (Bianchi and Phillips, 2005) measured the psychological resilience of undergraduates, and its negative association with CD-RISC was also reported by previous studies (Li and Guo, 2023; Xie et al., 2023). A higher MPAI represents a higher level of mobile phone addiction. Mobile phone overuse may disrupt the sleep process and lead to depression. Furthermore, long-term exposure to blue light and electromagnetic fields emitted from the screen may affect melatonin levels and contribute to poor sleep quality (Demirci et al., 2015). However, newer models of cell phones currently have the ability to turn off blue light, and this study did not investigate the condition with blue light turned off, so this finding may have limitations. A gender-specific study (Zhou et al., 2022) found that smoking was a risk factor for poor sleep quality among males, and noisy dormitory and academic pressure were risk factors for poor sleep quality among females. A study on college students of Jilin Province (Li Y. et al., 2020) found that drinking, academic pressure, and relationships with classmates were risk factors for poor sleep quality. A study on medical students (Shao et al., 2020) showed that students with greater employment pressure had more anxiety symptoms, which may affect their sleep quality. A cluster randomized-controlled trial in China (Li et al., 2022) showed that intervening with the sleep schedule of roommates can obtain a good sleep quality for them, suggesting that our findings of roommates sleeping late as a risk factor for poor sleep quality are consistent with it. However, there were some studies that considered low physical activity via the International Physical Activity Questionnaire-Short Form anxiety and depression scores via the Hospital Anxiety and Depression Scale as negative factors for sleep quality (Ghrouz et al., 2019). Another study (Nyer et al., 2013) found that students with depressive symptoms and sleep disturbance endorsed significantly more intense and frequent anxiety and poorer cognitive and physical functioning.

Being in a higher grade was a risk factor for sleep quality in weighted MLR and BLR, but only the random effects of being in the second grade and fourth grade or above were found to be positive in weighted LMM and GLMM, and the random effects of being in the first and third grades were negative. This may be due to the greater pressure faced by students in fourth grade or above, such as postgraduate entrance exams, employment, internships, and graduation thesis issues. Similarly, second grade was the year with the greatest learning pressure, and in addition to regular studies, they also needed to participate in various competitions and innovation training programs. The first grade was still in a transitional adaptation period from high school to college, while the third grade was a comfortable period after adapting to college life. The students in third grade were able to cope easily with previous pressure, and there was no new pressure similar to those in the fourth grade.

In the weighted LMM result, the slope of the relationship with classmates was positive, i.e., 0.812, in the subgroup of having no academic pressure, indicating that a poorer relationship with classmates meant a higher PSQI score. The corresponding slopes were negative, i.e., −0.458 and −0.353, in the subgroups of normal and great academic pressure, respectively, indicating that a poorer relationship with classmates meant a lower PSQI score. This may be because students who have good relationships with classmates need to spend more time dealing with communication or other issues between classmates, resulting in less rest time and high pressure. We recognize that this interpretation is speculative and based on observations from the current data set. Therefore, more comprehensive research is necessary to explore and test this hypothesis. Therefore, we call for further research to explore the complexity of the interplay between social stress, social relationships, and sleep quality.

In the weighted LMM result, the random intercept of having no employment pressure was 0.086, and its corresponding OR in the LRRE result was more than 1, which indicates that having no employment pressure had the effect of increasing the risk of poor sleep quality. This may be caused by the fact that the sample size of students with poor sleep quality and having no employment pressure was extremely low, i.e., 286. Thus, the model may not show the true effect of having no employment pressure. Another reason may be the random slopes of relationships with classmates, whose random effects were not significant.

The associated factors for sleep quality identified in various studies may differ, primarily due to several reasons. Firstly, the survey population and hierarchical structure may vary. Differences in population, region, and composition can yield diverse outcomes. Secondly, the included independent variables may differ; the same variable may have different values. Thirdly, the interaction between variables can lead to discrepancies in results. Furthermore, different models can yield different results. Even when analyzing data using the same model, different researchers may obtain distinct results due to differences in the operational methods of the model. The first two conditions are difficult to control, but we can try to avoid differences in results through standardized operations.

In the present study, four models were utilized to examine the factors associated with sleep quality. To ensure the robustness of the outcomes, we carried out rigorous diagnostics for all four models. After diagnostics, all four models obtained the best linear unbiased estimators (BLUE) or best linear unbiased predictions (BLUP) according to Gauss-Markov's theorem and Atkin's theorem (Hansen, 2022). Regression diagnostics are an important step that has been overlooked by many researchers. Standard diagnostics ensure more accurate parameter estimation and *p*-values and ultimately improve the overall quality of the analysis. Without diagnostics, some biased results for regression models will be obtained and reported. In the weighted MLR (resp., BLR) model, we found that other levels will increase or decrease PSQI scores (resp., risk of poor sleep quality) relative to the reference level. However, we can obtain detailed effect values of each level through LMM and LRRE. To our knowledge, previous studies have not shown similar results.

### Limitations

There are several limitations in the present study. Firstly, the present study selected as many candidate influencing factors as possible for sleep quality, such as family support, social support, personal lifestyle, physical health, mental health, mobile phone addiction, and psychological resilience. However, considering the length of the questionnaire, there were still some potential factors that were not included, such as coffee drinks, depression, and habits. Secondly, this study was cross-sectional, which precluded the establishment of definitive conclusions regarding the direction of causality between sleep quality and risk factors. Further, longitudinal studies are needed to investigate the causal relationships. Thirdly, all questionnaires were self-reported, highlighting the inherent limitations of self-reported measures. Lastly, there were no strict exclusion criteria in this study, which may have resulted in self-selection bias. This means that participants with anxiety and depression are characteristics that are likely to interact with the outcome under investigation, or alternatively, they may have been more likely to participate since the topic is relevant to them.

## Conclusion

This study examined the prevalence of risk factors associated with poor sleep quality among undergraduates in Jiangsu Province, China. Our results showed a considerable prevalence of poor sleep quality among this group, with students in higher grades exhibiting a higher likelihood of experiencing poor sleep quality. The study also identified modifiable factors that correlate with poor sleep quality, including psychological resilience, mobile phone addiction, smoking, drinking, and poorer physical health. In response to the factors associated with poor sleep quality identified in our study, we advocate for university administrators to deploy systematic educational programs and interventions tailored to enhance sleep quality among students. These initiatives should directly target modifiable risk factors such as mental health status, academic and employment pressures, and suboptimal dormitory conditions. Specifically, proposed interventions include organizing workshops and seminars on sleep hygiene to educate students about the importance of good sleep practices and the physiological underpinnings of sleep. University administrators should also offer mental health services that provide counseling and stress management strategies. They should facilitate sessions on academic and time management skills to mitigate the impact of academic and employment pressures and improve dormitory living conditions through the establishment of quiet hours and better noise insulation to foster a more sleep-conducive environment. These recommendations illustrate a thoughtfully considered approach to ameliorate the sleep-related challenges faced by university students. University administrators can provide adequate psychological counseling for students to alleviate their pressures and set appropriate dormitory conventions to address dormitory-related issues.

## Data availability statement

The original contributions presented in the study are included in the article/Supplementary material, further inquiries can be directed to the corresponding authors.

## Ethics statement

The studies involving humans were approved by Ethics Committee of Xuzhou Medical University. The studies were conducted in accordance with the local legislation and institutional requirements. The participants provided their written informed consent to participate in this study.

## Author contributions

BH: Methodology, Writing—review & editing. WS: Data curation, Funding acquisition, Writing—review & editing. YW: Writing—review & editing. QW: Data curation, Investigation, Writing—review & editing. JL: Writing—review & editing. XX: Writing—review & editing. YH: Writing—review & editing. LX: Data curation, Funding acquisition, Methodology, Writing—original draft. DY: Conceptualization, Writing—review & editing.

## Supplementary material

Supplementary Table S1 Pittsburgh Sleep Quality Index scores of undergraduates with different characteristics.

Supplementary Figure S1 The performance of linearity, homoscedasticity, extreme values, and normality after diagnostics for weighted multiple linear regression model.

Supplementary Figure S2 The performance of linearity and extreme values after diagnostics for binary logistic regression model.

Supplementary Figure S3 The performance of linearity, homoscedasticity, extreme values, and normality after diagnostics for the weighted linear mixed model.

Supplementary Figure S4 The performance of linearity after diagnostics for logistic regression with random effects model.

Supplementary Figure S5 The linear and quadratic trend between MPAI and PSQI score in weighted multiple linear regression.

Supplementary Figure S6 ROC curve of binary logistic regression model.

Supplementary Figure S7 The linear and quadratic trend between age and PSQI score in weighted linear mixed model.

Supplementary Figure S8 ROC curve of logistic regression with random effects model.
