# Supplementary material for: Prevalence and related factors of sleep quality among Chinese undergraduates in Jiangsu Province: multiple models' analysis
Source: Front Psychol. 2024 Apr 10;15:1343186. doi: 10.3389/fpsyg.2024.1343186 (PMC11040509; doi:10.3389/fpsyg.2024.1343186)
Supplement: Supplementary file 1 [file Data_Sheet_1.PDF]

Questionnaire on Sleep, Cell Phone Dependence, and Psychological Resilience among College Students in Jiangsu Province

Dear Friend:

Thank you for taking the time to fill out our questionnaire. Our research relies heavily on the valuable input and feedback from our participants. Your answers will help us gain insight into a particular topic or issue and provide valuable information for academic research. Please be as truthful as possible when answering the questions and expressing your views and opinions. Your personal information will be treated confidentially and will not be associated with your answers. If you are confused or unsure about something, please try to select the option that best fits your current situation. Each of your responses is critical to our research. Thank you again for your support. If you have any questions or need further clarification after completing the questionnaire, please feel free to contact us. Have fun filling out the questionnaire!

Thanks!

Sincere greetings.

Q1. What is your gender?

a. Male

b. Female

Q2. Your age: ___________________

Q3. Your height (m): ___________________

Q4. Your weight (Kg): ___________________

Q5. Location of your college or university: ___________________

Q6. What grade level are you in?

a. First year

b. Second year

c. Third year

d. Fourth year or more

Q7. Your specialty

a. Medical specialty

b. Non-medical specialty

Q8. Your native place

a. Urban

b. Rural

Q9. Only child or not?

a.Yes

b.No

Q10. Father's education level

a. Middle school or less

b. Junior college education

c. Undergraduate or more

Q11. Mother’s education level

a. Middle school or less

b. Junior college education

c. Undergraduate or more

Q12. What is your family's financial status?

a.Good

b.Medium

c.Poor

Q13. Your monthly cost of living

a.<1000

b.1000-2000

c.>2000

Q14. Your smoking status

a.Never

b.Seldom

c.Often

Q15. Your drinking status

a.Never

b.Seldom

c.Often

Q16. Your frequency of physical activity

a. ≤ 1 time per month

b. 1~3 times per week

c. 4-7 times per week

Q17. Your academic pressure situation

a.Not have

b.Normal

c.Great

Q18. Your employment pressure situation

a.Not have

b.Normal

c.Great

Q19. Dormitory Environment

	Yes	No
19.1 Do you think housemates sleep late?	○	○
19.2 Do you think the dormitory is noisy?	○	○
19.3 Do you think the dormitory lights are bright?	○	○
19.4 Do you approve of the hygiene of the dormitory?	○	○

Q20. How do you feel about relationships with your classmates?

a. Harmonious

b. Ordinary

c. Poor

Q21. Are you in love?

a.Never (Please skip to Q23)

b. Ever (Please skip to Q23)

c. Being in love

Q22. How is your relationship with your lover?

a. Harmonious

b. Ordinary

c. Poor

Q23. Your physical health status

a.Good

b.Ordinary

c.Bad

Q24. Your mental health status

a.Good

b.Ordinary

c.Bad

Q25. Do you think you need psychological counseling for your current condition?

a.Yes

b.No

Mobile phone addiction index (MPAI)

	Never	Seldom	Sometimes	Often	Always
1. Your friends and family have complained because you are on your cell phone	○	○	○	○	○
2. Someone said you spend too much time on your cell phone	○	○	○	○	○
3. You have tried to hide from others how much time you spend on your cell phone	○	○	○	○	○
4. Your phone bill is overspent	○	○	○	○	○
5. You find yourself using your cell phone for a longer period of time than originally intended	○	○	○	○	○
6. You try to spend less time on your phone but	○	○	○	○	○

can't					
7. You never feel like spending enough time on your phone	○	○	○	○	○
8. When staying outside the cell phone signal area for a while, you are always worried that you will miss the call	○	○	○	○	○
9. You'll have a hard time turning your cell phone off	○	○	○	○	○
10. If you haven't checked your texts in a while or your phone isn't on, you become anxious	○	○	○	○	○
11. You can't concentrate without your cell phone	○	○	○	○	○
12. If you don't have a cell phone, your friends will have a hard time reaching you	○	○	○	○	○
13. When feeling isolated, you use your cell phone to chat with others	○	○	○	○	○
14. When feeling lonely, you use your cell phone to chat with others	○	○	○	○	○
15. When depressed, you play with your cell phone to improve your mood	○	○	○	○	○
16. Finding yourself addicted to cell phone when you have other things you have to do and getting yourself in trouble for it	○	○	○	○	○
17. Time spent on cell phones leads to less efficient errands	○	○	○	○	○

Connor-Davidson Resilience Scale (CD-RISC)

	Never	Seldom	Sometimes	Often	Always
1. I can adapt to changes	○	○	○	○	○
2. I have close and secure relationships	○	○	○	○	○
3. Sometimes fate or God can help	○	○	○	○	○

4. I can handle whatever happens	○	○	○	○	○
5. Past successes have given me the confidence to face challenges	○	○	○	○	○
6. I can see the humorous side of things	○	○	○	○	○
7. Coping with stress makes me feel empowered	○	○	○	○	○
8. I tend to recover quickly after experiencing hardship or illness	○	○	○	○	○
9. Things always happen for a reason	○	○	○	○	○
10. I will do my best no matter what the outcome is	○	○	○	○	○
11. I can achieve my goals	○	○	○	○	○
12. I don't give up easily when things don't look promising	○	○	○	○	○
13. I know where to get help	○	○	○	○	○
14. Under pressure, I am able to concentrate and think clearly	○	○	○	○	○
15. I like to take the lead in solving problems	○	○	○	○	○
16. I will not be discouraged by failure	○	○	○	○	○
17. I consider myself to be a powerful person	○	○	○	○	○
18. I can make unusual or difficult decisions.	○	○	○	○	○
19. I can deal with unhappiness	○	○	○	○	○
20. I have to follow my instincts	○	○	○	○	○
21. I have a strong sense of purpose	○	○	○	○	○
22. I feel in control of my own life	○	○	○	○	○
23. I like challenges	○	○	○	○	○
24. I work hard to achieve my goals	○	○	○	○	○
25. I am proud of my accomplishments	○	○	○	○	○

Pittsburgh Sleep Quality Index (PSQI)

The following questions are about your sleep during the last month. Please select or fill in the answer that best describes your sleep during the last month.

Q1. What time did you usually go to bed at night? ___： ___

Q2. How long (in minutes) did it usually take you to fall asleep? __________________

Q3. What time did you usually get up each morning? ___： ___

Q4. How many hours of actual sleep have you gotten each night? __________________

Q5. How many times in the past month have you had the following sleep problems：

	Never	⩽ 1 time per week	1-2 times per week	≥ 3 times per week
5.1 Failure to sleep (within 30 minutes)	○	○	○	○
5.2 Waking up in the middle of the night or early	○	○	○	○
5.3 Going to toilet at night	○	○	○	○
5.4 Poor Breathing	○	○	○	○
5.5 Coughing or high snoring	○	○	○	○
5.6 Feeling cold	○	○	○	○
5.7 Feeling too hot	○	○	○	○
5.8 Nightmares	○	○	○	○
5.9 Feeling pain	○	○	○	○
5.10 Other things that affect sleep	○	○	○	○

Q6. You rated the quality of your sleep over the past month:

a. Very good

b. Good

c. Bad

d. Very bad

Q7. In the past month, your use of hypnosis with drugs

a. None

b. <1 time/week

c. 1-2 times/week

d. ⩾ 3 times/week

Q8. How often in the past month did you have difficulty staying awake while driving, eating or attending social events?

a. None

b. <1 time/week

c. 1-2 times/week

d. ⩾ 3 times/week

Q9. In the past month, have you had difficulty in maintaining enough energy to get things done?

a. No difficulty

b. A little difficult

c. Quite difficult

d. Very difficult
