# Supplementary material for: Prevalence and related factors of sleep quality among Chinese undergraduates in Jiangsu Province: multiple models' analysis
Source: Front Psychol. 2024 Apr 10;15:1343186. doi: 10.3389/fpsyg.2024.1343186 (PMC11040509; doi:10.3389/fpsyg.2024.1343186)
Supplement: Supplementary Table S1 — Pittsburgh Sleep Quality Index scores of undergraduates with different characteristics. [file Table_1.docx]

**Supplementary materials for “Prevalence and related factors of sleep quality among Chinese undergraduates in Jiangsu Province: Multiple models’ analysis”**

**Table S1** Pittsburgh Sleep Quality Index scores of undergraduates with different characteristics

| Variables | *n* (%) | PSQI, mean±SD | P |
| --- | --- | --- | --- |
| **Gender** |  |  | 0.1960 |
| Female | 5917 (70.00) | 5.67±3.51 |  |
| Male | 2540 (30.00) | 5.56±3.80 |  |
| **Grade** |  |  | **<0.001** |
| First year | 5140 (60.79) | 5.32±3.47 | a |
| Second year | 2496 (29.50) | 6.11±3.75 | b |
| Third year | 357 (4.22) | 6.00±3.58 | b |
| Fourth year or more | 464 (5.49) | 6.33±3.83 | b |
| **Medical Major** |  |  | 0.058^#^ |
| No | 7233 (85.50) | 5.67±3.62 |  |
| Yes | 1224 (14.50) | 5.46±3.52 |  |
| **Native place** |  |  | **0.028**^#^ |
| Urban | 3350 (39.60) | 5.71±3.55 |  |
| Rural | 5107 (60.40) | 5.53±3.68 |  |
| **Only child or not** |  |  | **0.006**^#^ |
| No | 5187 (61.30) | 5.72±3.58 |  |
| Yes | 3270 (38.70) | 5.5±3.63 |  |
| **Father’s education level** |  |  | **0.001** |
| Middle school or less | 5834 (69.00) | 5.73±3.57 | a |
| Junior college education | 1369 (16.20) | 5.38±3.43 | b |
| Undergraduate or more | 1254 (14.80) | 5.46±3.91 | a, b |
| **Mother’s education level** |  |  | **0.041**^#^ |
| Middle school or less | 6256 (74.00) | 5.69±3.57 | a |
| Junior college education | 1229 (14.50) | 5.48±3.61 | a |
| Undergraduate or more | 972 (11.50) | 5.46±3.83 | a |
| **Family economic level** |  |  | **<0.001** |
| Good | 725 (8.57) | 5.12±3.73 | a |
| Medium | 6619 (78.27) | 5.56±3.51 | b |
| Poor | 1113 (13.16) | 6.44±3.92 | c |
| **Monthly living expense** |  |  | **<0.001** |
| <1000 | 1030 (12.20) | 6.25±3.94 | a |
| 1000-2000 | 6205 (73.40) | 5.52±3.48 | b |
| >=2000 | 1222 (14.40) | 5.71±3.85 | b |
| **Smoking** |  |  | **<0.001** |
| No | 8027 (94.91) | 5.55±3.53 |  |
| Yes | 430 (5.09) | 7.24±4.49 |  |
| **Drinking** |  |  | **<0.001** |
| No | 5775 (68.28) | 5.38±3.48 |  |
| Yes | 2682 (31.72) | 6.18±3.79 |  |
| **Physical exercise** |  |  | **<0.001** |
| ≤1 per month | 1391 (16.40) | 6.36±3.94 | a |
| 1-3times per week | 5681 (67.20) | 5.57±3.50 | b |
| 4-7times per week | 1385 (16.40) | 5.19±3.59 | c |
| **Academic pressure** |  |  | **<0.001** |
| No | 378 (4.40) | 4.67±4.00 | a |
| Normal | 6052 (71.60) | 5.33±3.39 | b |
| Great | 2027 (24.00) | 6.72±3.90 | c |
| **Employment pressure** |  |  | **<0.001** |
| No | 1421 (16.80) | 4.90±3.45 | a |
| Normal | 4281 (50.60) | 5.33±3.39 | b |
| Great | 2755 (32.60) | 6.49±3.83 | c |
| **Relationship with classmates** |  |  | **<0.001** |
| Harmonious | 5877 (69.50) | 5.23±3.45 | a |
| Ordinary | 2453 (29.00) | 6.46±3.63 | b |
| Poor | 127 (1.50) | 8.57±5.41 | c |
| **Amativeness matter** |  |  | **0.0420** |
| Never | 4059 (48.00) | 5.53±3.56 | a |
| Ever | 2745 (32.50) | 5.73±3.61 | b |
| Being in love | 1653 (19.50) | 5.73±3.69 | c |
| **Physical health status** |  |  | **<0.001** |
| Good | 3668 (43.40) | 4.64±3.24 | a |
| Ordinary | 4395 (52.00) | 6.21±3.50 | b |
| Bad | 394 (4.60) | 8.5±4.81 | c |
| **Mental health status** |  |  | **<0.001** |
| Good | 4209 (49.80) | 4.56±3.17 | a |
| Ordinary | 3787 (44.80) | 6.45±3.49 | b |
| Bad | 461 (5.40) | 8.75±4.57 | c |
| **Psychological counseling** |  |  | **<0.001** |
| No | 7490 (88.60) | 5.41±3.46 |  |
| Yes | 967 (11.40) | 7.37±4.15 |  |

Note: # denotes that the groups satisfy homoscedasticity and others does not. Letters a, b, and c represents the difference between intergroups. If the cells contain the same letters, it means there is no difference between the two intergroups. P values less than 0.05 are given in bold. For Mother’s education level group, Welch’s ANOVA gave significant result, but Tukey’s, Student-Newman-Keuls’ (SNK), Bonferroni’s, Scheffe’s and Holm’s test were all gave non-significant results for post hoc. The PSQI score did not use median to describe its central tendency because median cannot show the differences among subgroups.


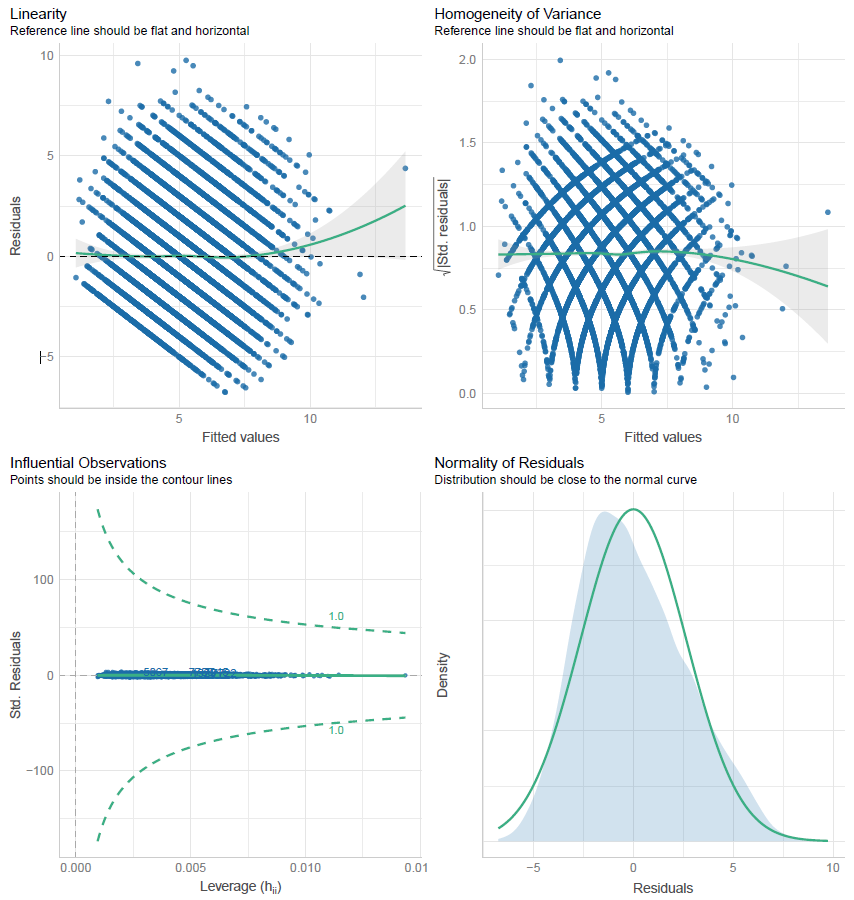


**Figure S1** The performance of linearity, homoscedasticity, extreme values, and normality after diagnostics for weighted multiple linear regression model


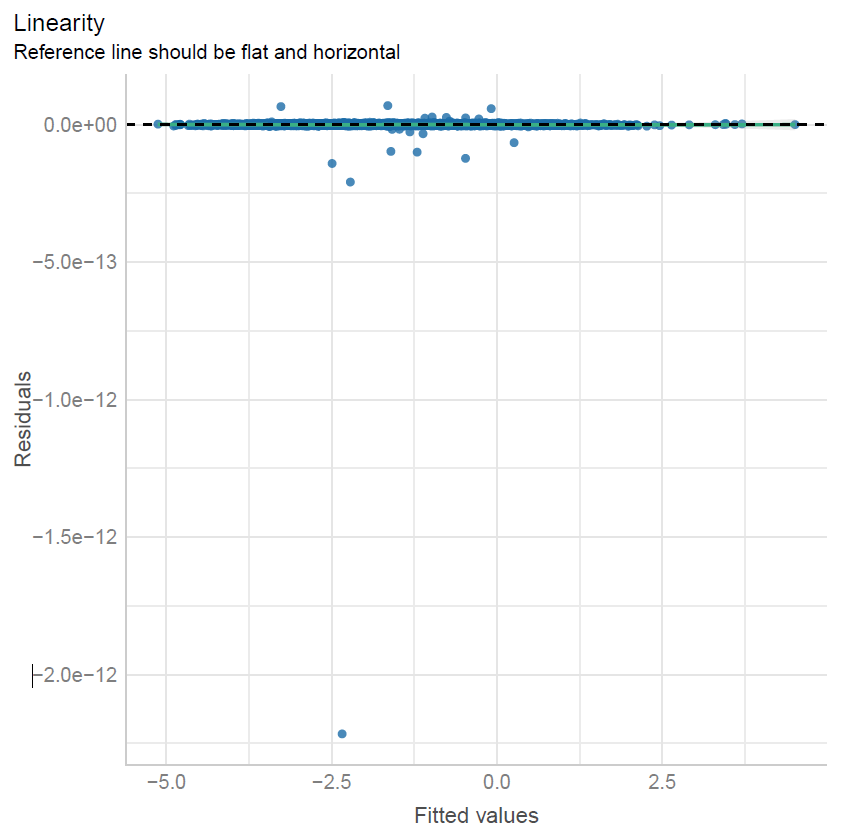

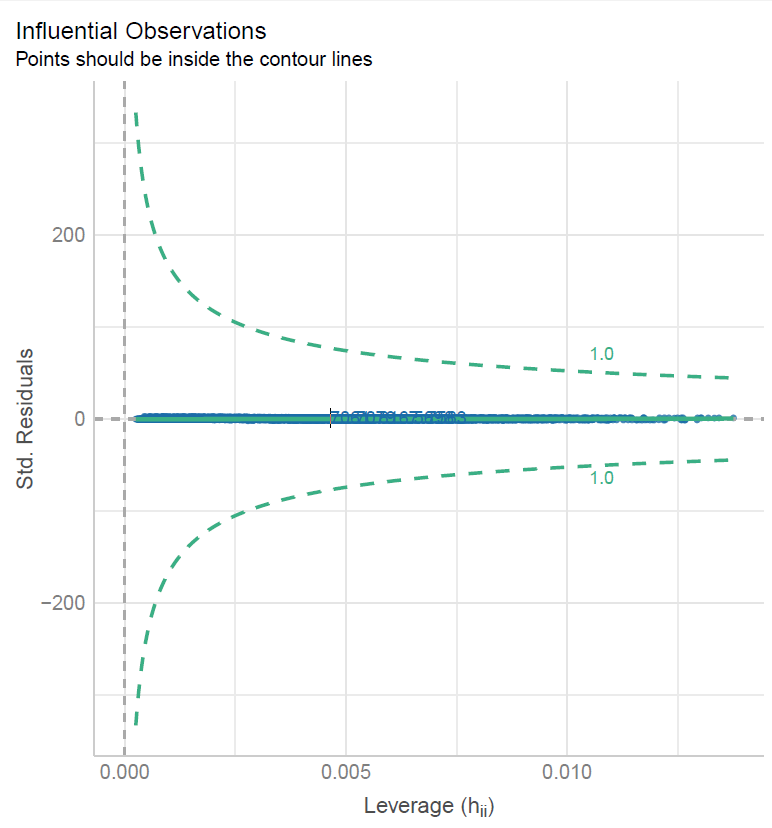


**Figure S2** The performance of linearity and extreme values after diagnostics for binary logistic regression model


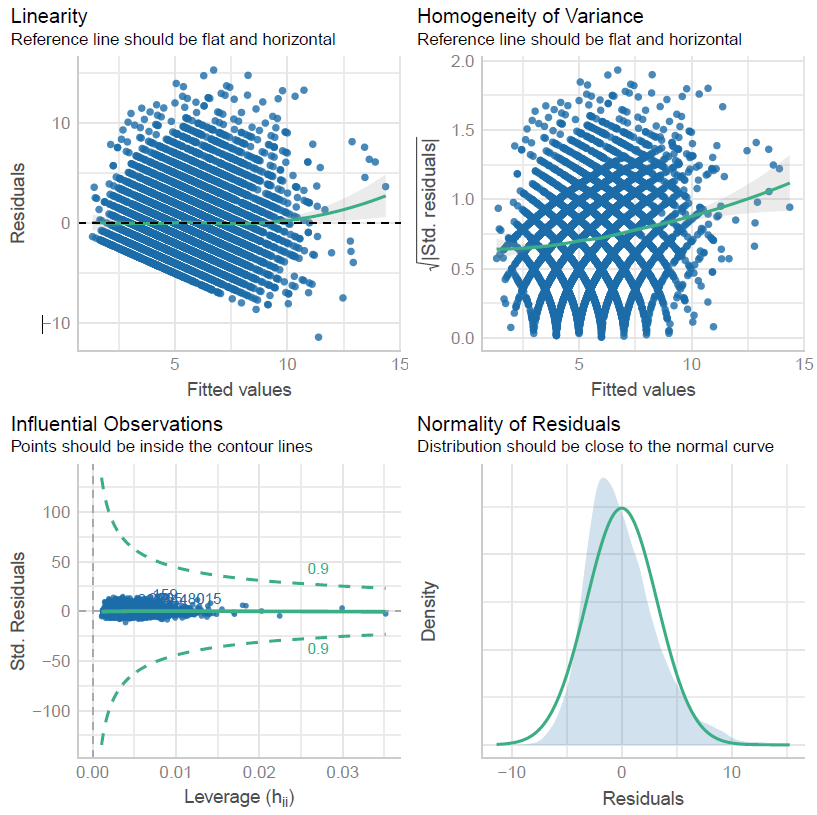


**Figure S3** The performance of linearity, homoscedasticity, extreme values, and normality after diagnostics for weighted linear mixed model


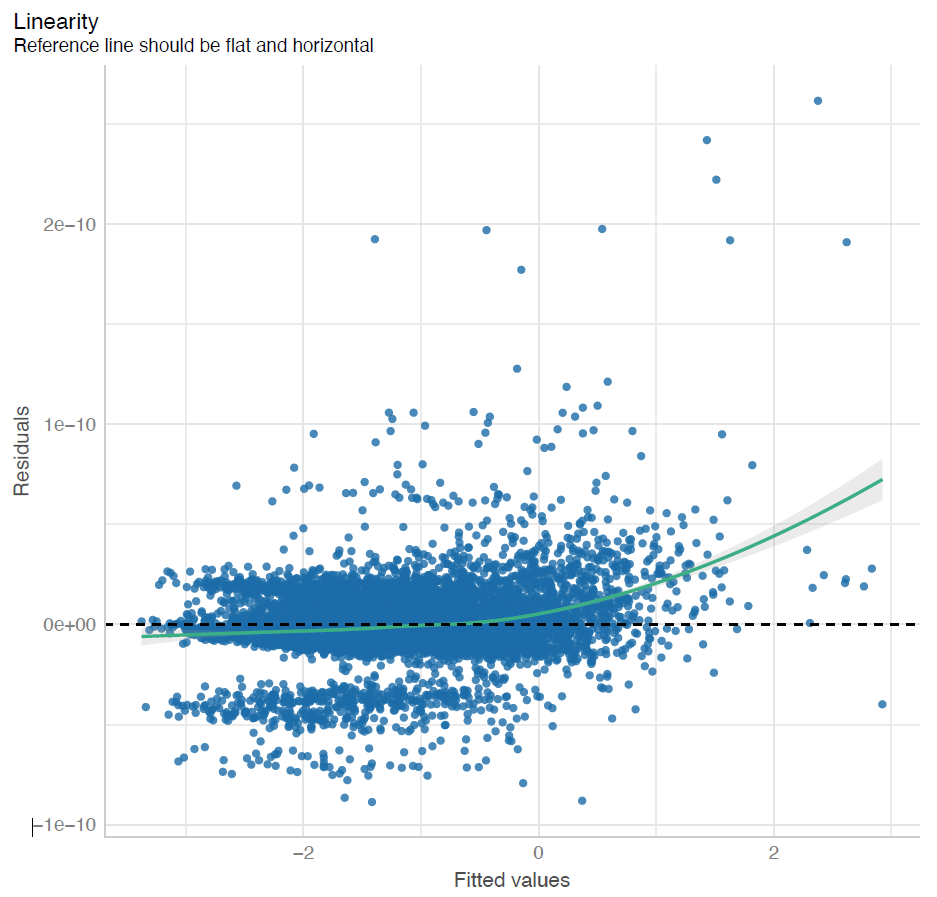


**Figure S4** The performance of linearity after diagnostics for logistic regression with random effects model


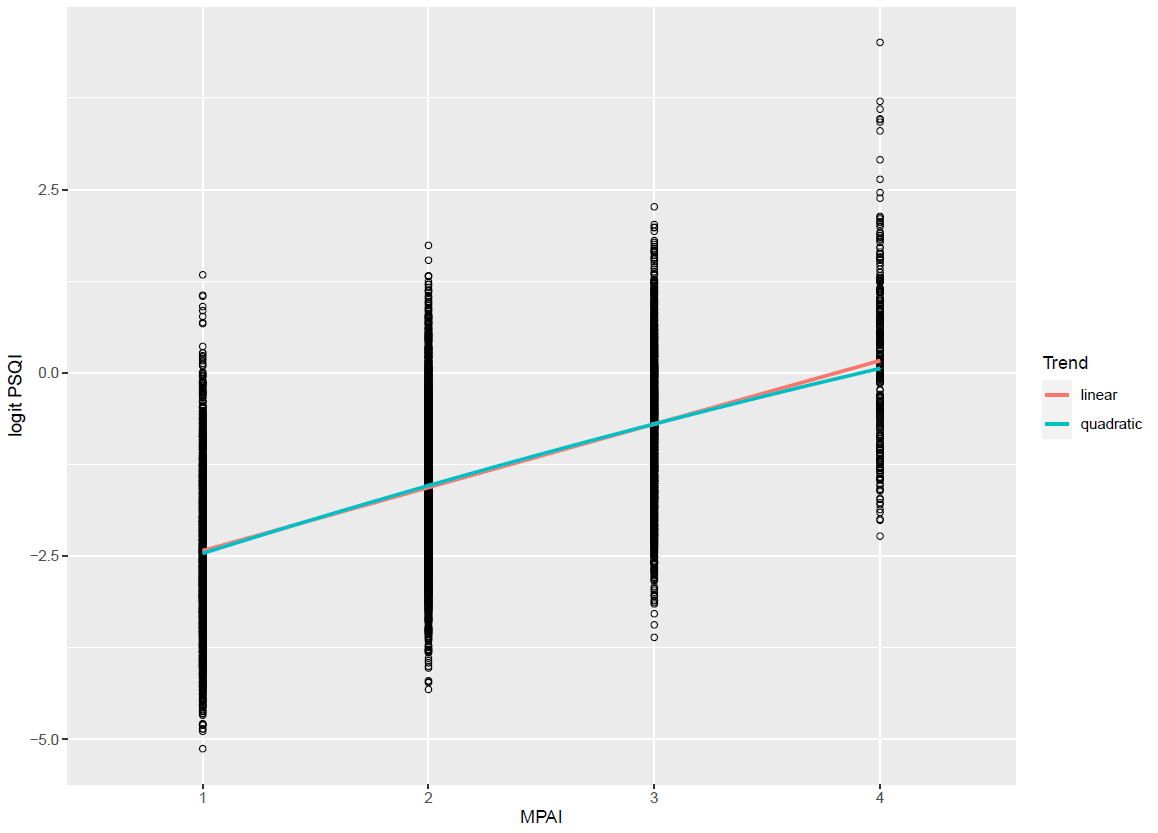


**Figure S5** Linear and quadratic trend between MPAI and PSQI score in weighted multiple linear regression


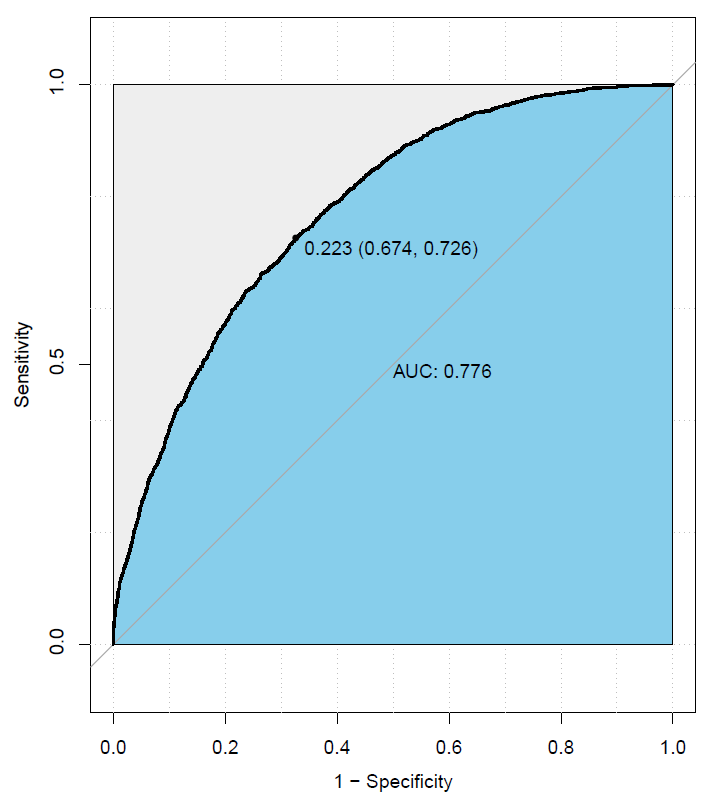


**Figure S6** ROC curve of binary logistic regression model


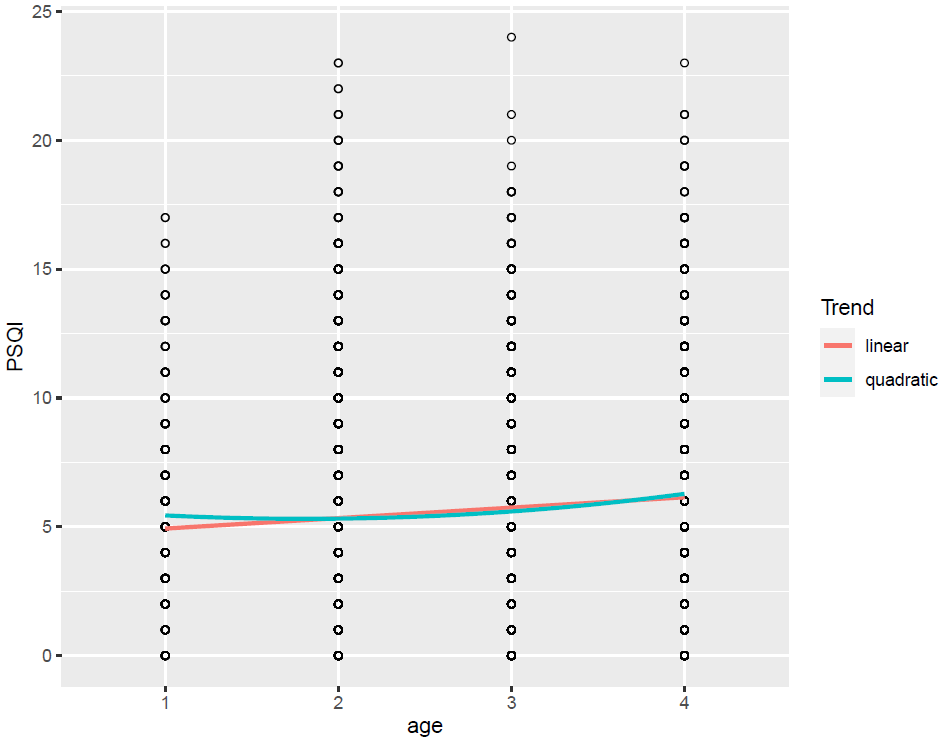


**Figure S7** Linear and quadratic trend between age and PSQI score in weighted linear mixed model


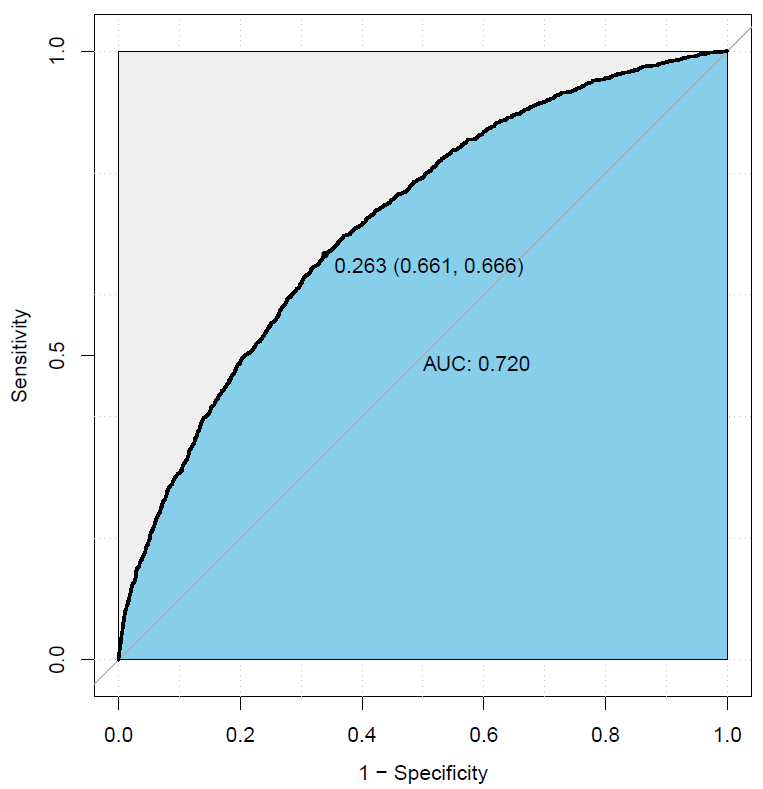


**Figure S8** ROC curve of logistic regression with random effects model
